# Correction: Engineering microbial physiology with synthetic polymers: cationic polymers induce biofilm formation in *Vibrio cholerae* and downregulate the expression of virulence genes

**DOI:** 10.1039/c8sc90189a

**Published:** 2018-09-27

**Authors:** Nicolas Perez-Soto, Lauren Moule, Daniel N. Crisan, Ignacio Insua, Leanne M. Taylor-Smith, Kerstin Voelz, Francisco Fernandez-Trillo, Anne Marie Krachler

**Affiliations:** a School of Biosciences , University of Birmingham , Edgbaston , B15 2TT Birmingham , UK; b Institute of Microbiology and Infection , University of Birmingham , Edgbaston , B15 2TT Birmingham , UK . Email: f.fernandez-trillo@bham.ac.uk; c School of Chemistry , University of Birmingham , Edgbaston , B15 2TT Birmingham , UK; d Department of Microbiology and Molecular Genetics , University of Texas McGovern Medical School at Houston , Houston , TX 77030 , USA . Email: anne.marie.krachler@uth.tmc.edu

## Abstract

Correction for ‘Engineering microbial physiology with synthetic polymers: cationic polymers induce biofilm formation in *Vibrio cholerae* and downregulate the expression of virulence genes’ by Nicolas Perez-Soto *et al.*, *Chem. Sci.*, 2017, **8**, 5291–5298.



## 

The *Vibrio cholerae* strain used in this work is A1552^1^ and not N16961. Both strains are O1 Inaba serotype and the main conclusions of the paper remain unchanged.

The Royal Society of Chemistry apologises for these errors and any consequent inconvenience to authors and readers.
