# Supplementary material for: Engineering microbial physiology with synthetic polymers: cationic polymers induce biofilm formation in Vibrio cholerae and downregulate the expression of virulence genes
Source: Chem Sci. 2017 May 16;8(8):5291–8. doi: 10.1039/c7sc00615b (PMC5607900; doi:10.1039/c7sc00615b)
Supplement: Supplementary file 1 [file SC-008-C7SC00615B-s001.pdf]

Electronic Supplementary Material (ESI) for Chemical Science.
This journal is © The Royal Society of Chemistry 2017

Engineering Microbial Physiology with Synthetic Polymers: Cationic Polymers Induce Biofilm Formation in Vibrio cholerae and Downregulate the Expression of Virulence Genes.

Nicolas Perez-Soto[a,b], Lauren Moule[a,b], Daniel N. Crisan[b,c], Ignacio Insua[b,c], Leanne M. Taylor-Smith[a,b], Kerstin Voelz[a,b], Francisco Fernandez-Trillo*[b,c] and Anne-Marie Krachler.[*a,b,d]

[a] School of Biosciences, [b] Institute of Microbiology and Infection, [c] School of Chemistry, University of Birmingham, Edgbaston, B15 2TT Birmingham, UK, E-mail: f.fernandez-trillo@bham.ac.uk
[d] Department of Microbiology and Molecular Genetics, University of Texas McGovern Medical School at Houston, Houston, TX, 77030, USA. E-mail: anne.marie.krachler@uth.tmc.edu.

Table of Contents

1. MATERIALS 2
2. MEDIA 2
3. INSTRUMENTATION 2
4. SYNTHESIS OF POLYMERS 3
4.1. SYNTHESIS AND CHARACTERISATION OF P(DMAPMAM) (P1). 3
4.2. SYNTHESIS AND CHARACTERISATION OF P(APMAM) (P2) 3
5. BACTERIAL STRAINS AND CULTURE CONDITIONS 4
6. PLASMID CONSTRUCTION 5
7. CLUSTERING 5
7.1. IMAGING OF BACTERIA CLUSTERS 5
8. MOTILITY ASSAY 6
9. EFFECT OF POLYMERS ON MICROBIAL GROWTH 6
9.1. GFP EXPRESSION 6
9.2. MICROBIAL MEMBRANE INTEGRITY 7
10. VIRULENCE 7
10.1. QUANTIFICATION OF BIOFILM FORMATION 7
10.2. IMAGING OF BIOFILM FORMATION AND QUANTIFICATION OF EXTRACELLULAR DNA 7
10.3. TRANSCRIPTIONAL ASSAYS: Β-GALACTOSIDASE ACTIVITY 8
11. COLONISATION AND TOXICITY OF CACO-2 CELLS 9
11.1. COLONY FORMING UNITS 9
11.2. CYTOTOXICITY IN CACO-2 CELLS: LDH ASSAY 9
11.3. CYTOTOXICITY IN CACO-2 CELLS: CAMP ASSAY 10
11.4. IMAGING OF CACO-2 CELLS 11

12. COLONISATION OF ZEBRAFISH 11

12.1. ZEBRAFISH CARE AND MAINTENANCE 11
12.2. COLONY FORMING UNITS 11
12.3. IMAGING OF ZEBRAFISH LARVAE 12

13. REFERENCES 12

1. Materials

N-(3-aminopropyl)methacrylamide hydrochloride (APMAm), *N*-[3-(dimethylamino)propyl]methacrylamide (DMAPMAm), 2,2'-azobis(2-methylpropionitrile) (AIBN) and Dulbecco's Modified Eagle's Medium (DMEM) were purchased from Sigma-Aldrich. 4,4'-azobis(4-cyanovaleric acid) (ACVA) and 2-mercaptoethanol were bought from Alfa Aesar. Polyethylene glycol standards were purchased from Agilent Technologies. All other chemicals were purchased from Fisher Scientific and VWR and were used without further purification. Polymers were synthesised by radical polymerisation as described below.

2. Media

- **LB**: 10 g/L tryptone, 10 g/L NaCl and 5 g/L yeast extract
- **DMEM**: 0.265 g/L $CaCl_2$, 0.0001 g/L $Fe(NO_3)_3$, 0.09767 g/L $MgSO_4$, 0.4 g/L KCl, 3.7 g/L $NaHCO_3$, 6.4 g/L NaCl, 0.109 g/L NaH_2PO_4, 0.084 g/L L-Arg, 0.0626 g/L L-Cys, 0.03 g/L Gly, 0.042 g/L L-His, 0.105 g/L L-Iso, 0.105 g/L L-Leu, 0.146 g/L L-Lys, 0.03 g/L L-Met, 0.066 g/L L-Phe, 0.042 g/L L-Ser, 0.095 g/L L-Thr, 0.016 g/L L-Trp, 0.12037 g/L L-Tyr, 0.094 g/L L-Val, 0.004 g/L choline chloride, 0.004 g/L folic acid, 0.0072 *myo*-inositol, 0.004 g/L niacinamide, 0.00404 g/L pyridoxine, 0.004 g/L D-pantothenic acid, 0.0004 g/L riboflavin, 0.004 g/L thiamine, 4.5 g/L glucose and 0.584 g/L L-Glu
- **Red DMEM**: Same formulation plus 0.0159 g/L phenol red.
- **E3 media**: 0.292 g/L NaCl, 0.013 g/L KCl, 0.044 g/L $CaCl_2$, 0.081 g/L $MgSO_4$, 0.477 g/L HEPES pH 7.0

3. Instrumentation

Polymers were characterised by Nuclear Magnetic Resonance (NMR) and Gel Permeation Chromatography (GPC). NMR spectra were recorded on a Bruker Avance III spectrometer operating at 300 MHz and fitted with a 5 mm BBFO probe. Chemical shifts are reported in ppm (δ) referenced to the corresponding solvent signals: DMSO-*d6* (δ = 2.50) and D2O (δ = 4.79). GPC was recorded on a Shimadzu Prominence LC-20A, fitted with Shodex Asaphipak GF-510 HQ (300 × 7.5 mm, 5 µm) and GF-310 HQ (300 × 7.5 mm, 5 µm) columns in series, and equipped with a Thermo Fisher Refractomax 521 detector. GPC analysis was carried out using 100 mM acetate buffer at pH 2.9 as eluent at 40 °C and a flow rate of 0.6 $mL{\cdot}min^{-1}$. Molecular weights were calculated based on a standard calibration method using polyethylene glycol. Optical microscopy of *V. cholerae* clusters was measured on a Nikon-Eclipse TE2000-U microscope and Plan Apo 60x/ 1.40 NA oil DIC objective (Nikon) and captured with QICAM Fast1394 camera (Q imaging). Absorbance at 402 nm or 595 nm was recorded on a FLUOstar Omega plate reader. Absorbance at 420 nm was recorded using a Jenway 6305/UV Visible spectrophotometer. GFP fluorescence of GFP expressing *V. cholerae* cultures was recorded a FLUOstar Omega plate reader. Fluorescent-activated cell sorting (FACS) was performed on an Attune® Acoustic Focusing Cytometer (Applied BiosystemsTM). Agar plates were imaged using a ChemiDoc MP System (Biorad). Biofilms were imaged using a Nikon TE2000-U microscope using a 100x Plan Apo objective (Nikon) and captured with Digital Sight DS-Qi1MC camera (Nikon). Stained *V. cholerae* clusters were imaged using a Zeiss Axio Observer.Z1 microscope, Zeiss 40x/1.4 Plan Apochromat objective, ORCA-Flash4.0 camera (Hamamatsu) and ZEN 2.0.0.10 software. Stained Caco-2 cultures and Zebrafish larvae were imaged using a Zeiss Axio Observer.Z1 microscope with 63x/1.4 Plan Apochromat objective, ORCA-Flash4.0 camera (Hamamatsu) and ZEN 2.0.0.10 software. Spinning disk

was performed on a Nikon Eclipse-Ti microscope with CSU-X1 spinning Disk Confocal, Plan Apo VC 60x 1.4 NA Oil objective, Andor Neo CMOS camera and 488 nm excitation laser using Nikon Elements software for image acquisition.

4. Synthesis of Polymers

4.1. Synthesis and characterisation of p(DMAPMAm) (P1).

N-[3-(dimethylamino)propyl]methacrylamide (DMAPMAm) (2.2 mL, 12.025 mmol), 2,2'-azobis(2-methylpropionitrile) (AIBN) (19.6 mg, 0.117 mmol) and 2-mercaptoethanol (4.0 µL, 0.056 mmol) were dissolved in toluene (9.5 mL). This solution was degassed under argon for 10 minutes and then heated at 70 °C under stirring for 18 hours. After this time, the reaction flask was opened to the air and the crude was precipitated twice: first into diethyl ether (200 mL) and then into a diethyl ether/ hexane mixture (1:1) (100 mL). The precipitate was freeze-dried and a crystalline white solid was obtained (1.66 g, 87% yield). ^{1}H-NMR (300 MHz, DMSO-*d6*) δ (ppm): 7.51 (br, 1H, CO-NH), 2.96 (br, 2H, CO-NH-<u>CH$_2$</u>), 2.19 (br, 5H, <u>CH$_2$</u>-N-$(CH_3)_2$ + $CH_{3\ backbone}$), 2.11 (br, 6H, N-$(CH_3)_2$), 1.50 (br, 2H, <u>CH$_2$</u>-CH_2-N), 0.78 (br, 2H, $CH_{2\ backbone}$). Mn (buffer GPC) 46331, $Đ_M$ (buffer GPC) 1.14.

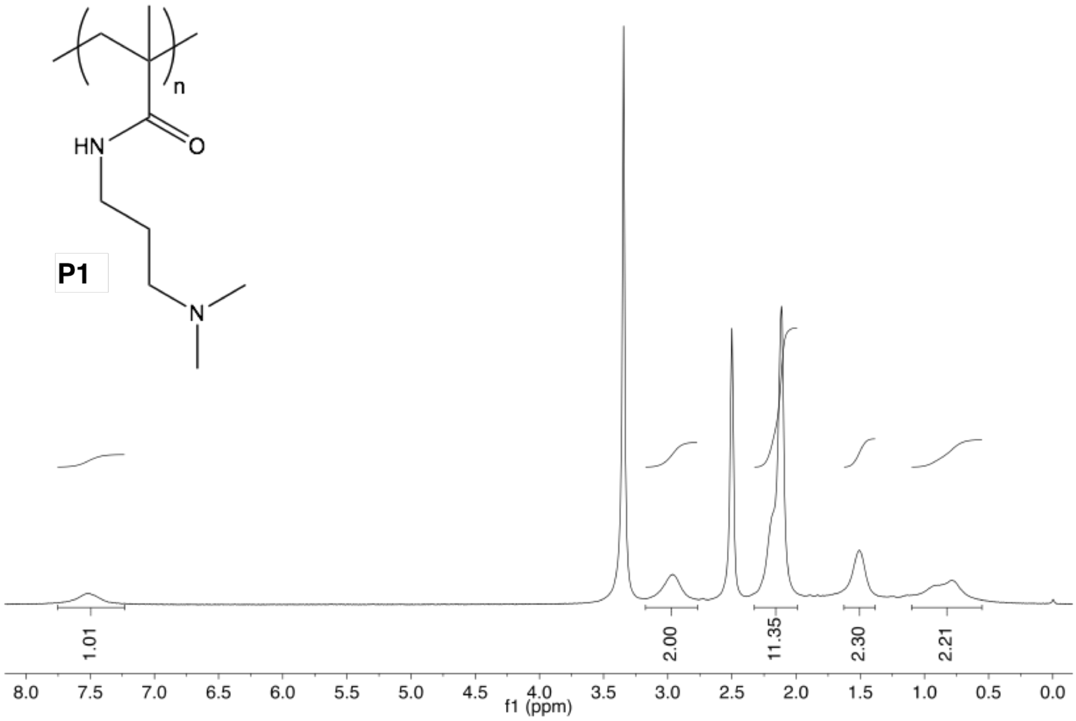

Fig. S1. ^{1}H-NMR (300 MHz, dmso-*d6*) spectrum of **P1**.

4.2. Synthesis and characterisation of p(APMAm) (P2)

N-(3-aminopropyl)methacrylamide (APMAm) hydrochloride (505.0 mg, 2.770 mmol), 4,4'-azobis(4-cyanovaleric acid) (ACVA) (12.4 mg, 0.033 mmol) and 2-mercaptoethanol (1.0 µL, 0.014 mmol) were dissolved in MilliQ water (2.2 mL). This solution was degassed under argon for 10 minutes and then heated at 70 °C under stirring for 17 hours. After this time, the reaction flask was opened to the air and the crude was precipitated three times into diethyl ether (50 mL). The precipitate was freeze-dried and a crystalline white solid was obtained (70.0 mg, 14% yield). ^{1}H-NMR (300 MHz, D_2O) δ (ppm): 3.11 (br, 3H, $CH_{3\ backbone}$), 2.93 (br t, *J*= 7.0 Hz, 2H, CO-NH-<u>CH$_2$</u>), 1.78 (br, 2H, -C<u>H$_2$</u>-NH_2), 1.63 (br, 2H, -C<u>H$_2$</u>-CH_2-NH_2), 0.98 (br, 1H, $CH_{2\ backbone}$), 0.83 (br, 1H, $CH_{2\ backbone}$). Mn (GPC) 46997, $Đ_M$ (GPC) 1.16.

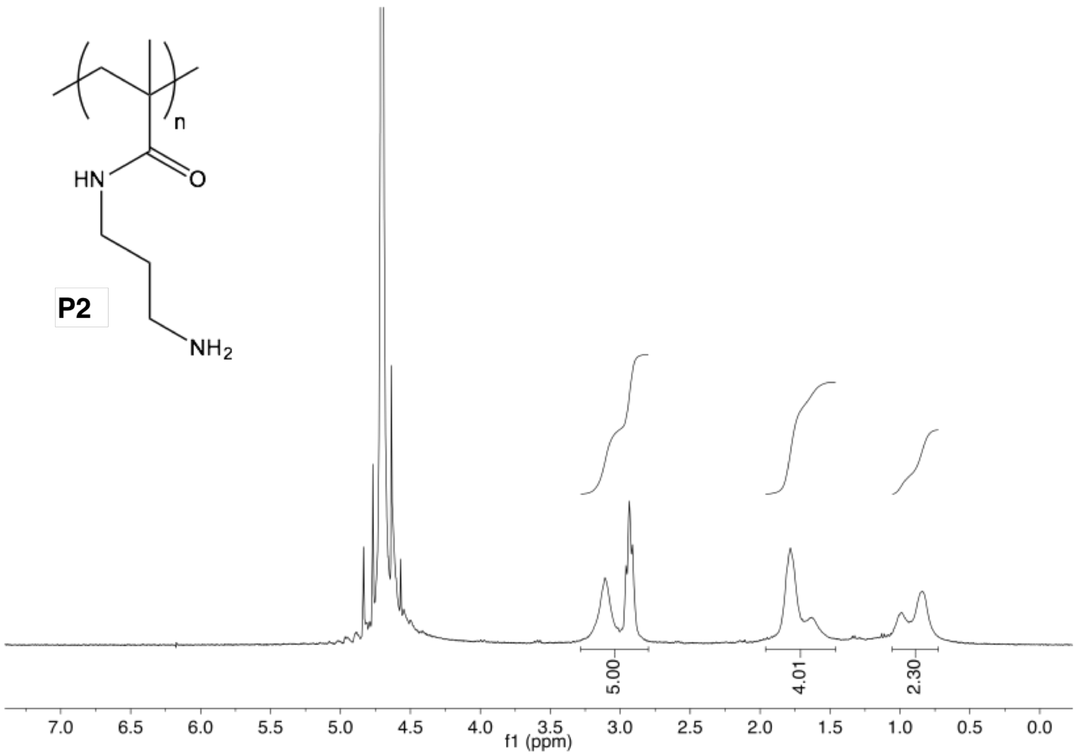

Fig. S2. ^{1}H-NMR (300 MHz, D_2O) spectrum of **P2**.

5. Bacterial strains and culture conditions

Vibrio cholerae strains used in this study (**Table S1**) were derived from the El Tor strain N16961 used as parental strain. Strains were propagated at 37 °C in Luria-Bertani (LB) broth supplemented with 50 µg/µL spectinomycin, 30 µg/µL kanamycin or 10 µg/µL tetracycline where selection was required. Plasmids were introduced into *V. cholerae* by conjugation, as previously described.[1] Briefly, aliquots of overnight cultures of *V. cholerae* N16961, *E. coli* DH5a carrying the desired plasmid (donor) and an *E. coli* SM10 helper strain carrying pRK2013 were mixed at a volumetric ratio of 1:2:2 and spotted onto brain-heart infusion (BHI) agar. Following overnight incubation, spots of bacterial growth were dislodged and suspended in 3 mL of PBS. 100 µL of bacterial suspension were plated onto M9 media containing 50 µg/µL of spectinomycin. Resulting colonies were checked by PCR and sequencing in the case of pRW50-oriT constructs, and by screening for green fluorescence in the case of pMW-*gfp* transformants.

Table S1. *V. cholerae* strains used in this study.

Strain	Description	Source or reference
N16961	Wild-type; O1 biovar El Tor.	Heidelberg et al.[2]
NP5001	N16961 carrying promoterless pRW50-oriT plasmid; Tet^R.	This study
NP5002	N16961 carrying pRW50-oriT plasmid containing the upstream region of *toxT* promoter; Tet^R.	This study
NP5003	N16961 carrying pRW50-oriT plasmid containing the upstream region of *ctxAB* promoter; Tet^R.	This study
NP5004	N16961 carrying pRW50-oriT plasmid containing the upstream region of *tcpA* promoter; Tet^R.	This study
NP5005	N16961 carrying pRW50-oriT plasmid containing the upstream region of *aphA* promoter; Tet^R.	This study

NPMW1	N16961 carrying pMW-*gfp* plasmid; Spect^R.	Ritchie et al.[3]
BH1651	*luxO*^{D47E}	Ng et al.[4]

6. Plasmid construction

Primers used for cloning and sequencing the constructs used in this study are listed in **Table S2** and were designed based on the sequence of *V. cholerae* N16961.[2] Primers were paired as appropriate to amplify upstream regions of *toxT*, *ctxAB*, *tcpA*, and *aphA* (**Fig. 4A**). Amplified DNA was digested using EcoR1 and HindIII, and ligated into digested pRW50-oriT vector (gift from the Grainger lab, University of Birmingham http://graingerlab.com). The vector is a derivative of pRW50,[5] which has been modified by inserting the *oriT* sequence from the vector pRK2.[6] The resulting reporter constructs were used to transform *V. cholerae* and the resulting strains are listed in **Table S1**.

Table S2 Primers used in this study, with restriction sites underlined.

Primer	Sequence (5´-3´)
pRW50 F	GTTCTCGCAAGGACGAGAATTTC
pRW50 R	AATCTTCACGCTTGAGATAC
aphApF1	TGCAGAATTCCTGGTTAACAAATCGCTAAATGTCAG
aphApR1	ATTCAAGCTTGTGTGGTAATGACATGTCTTCAATC
toxTpF1	TGTAGAATTCGATAAGATAACAGCCATATTCGTGG
toxTpR1	GATCAAGCTTTCCCAATCATTGCGTTCTACTC
ctxABpF1	GCTTGAATTCCTGTGGGTAGAAGTGAAACGG
ctxABpR1	TCATAAGCTTTATCTTTACCATATAATGCTCCCTTTG
tcpApF1	CTTAGAATTCGGTCTTATCATGAGCCGCC
tcpApR1	TGATAAGCTTTGCATATTTATATAACTCCACCATTTGTG

7. Clustering

7.1. Imaging of bacteria clusters

An overnight culture of *V. cholerae* (as described in section 5) was diluted to an OD_{600} of 1.0 in DMEM without phenol red and polymers were added to final concentrations ranging from 0-500 µg/mL (**Fig. 1** and **Fig. S3**). For imaging, aliquots of these cultures were taken after 15 and 60 min of incubation and mounted with ProLong® Antifade Gold solution (LifeTechnologies). Cured samples were visualised using a Nikon-Eclipse TE2000-U microscope and Plan Apo 60x/ 1.40 NA oil DIC objective (Nikon) and captured with QICAM Fast1394 camera (Q imaging). Representative images were taken using Nikon NIS-Elements software and prepared with ImageJ and Corel Draw X5 software.

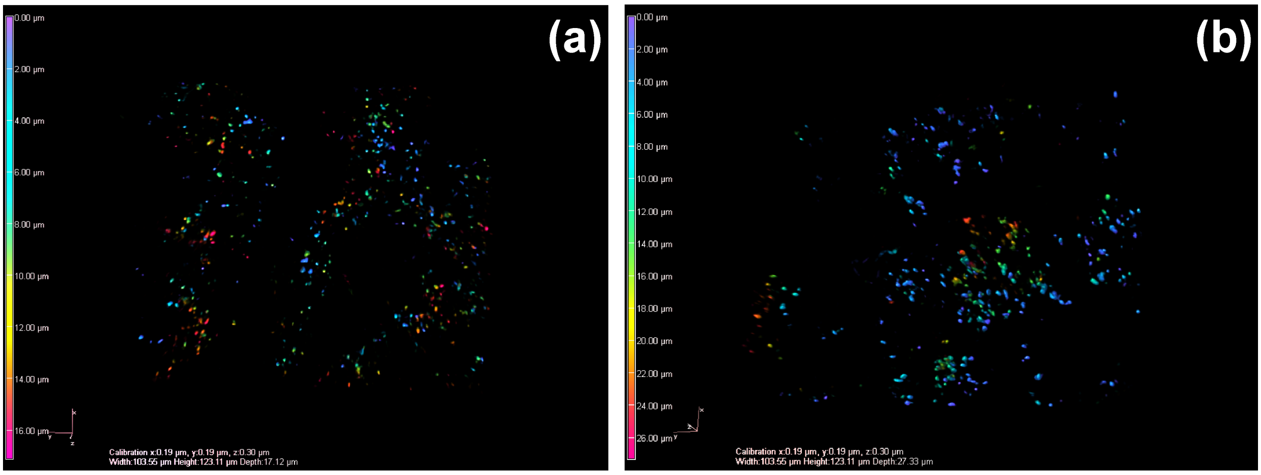

Fig. S3. Spinning disk fluorescent micrograph of GFP-*V. cholerae* N16967 incubated in PBS (pH 7.4) for 15 min in the presence 500 µg/mL of **P1 (a)** or **P2 (b)**. Maximum intensity projections are shown, with color-coded Z-depth. Total depth of the clusters shown here are 17 and 27 µm, respectively.

8. Motility assay

Motility of *V. cholerae* was assessed as previously described.[7] Briefly, overnight cultures of *V. cholerae* N16961 (as described in section 5) were diluted to an OD_{600} of 1.0 and then incubated in the absence and presence of polymers overnight. An aliquot of 1 µL was then placed into soft agar LB plates (0.4% agar) and incubated overnight at room temperature. For controls in the presence of high concentrations of salt, following incubation with polymers, samples were treated with 1 mL of PBS buffer containing 200 mM NaCl. When the solution turned cloudy an aliquot of 1 µL was placed into soft agar plates and incubated overnight at room temperature. After incubation, pictures of the plates were taken using a ChemiDoc MP System (Bio-rad).

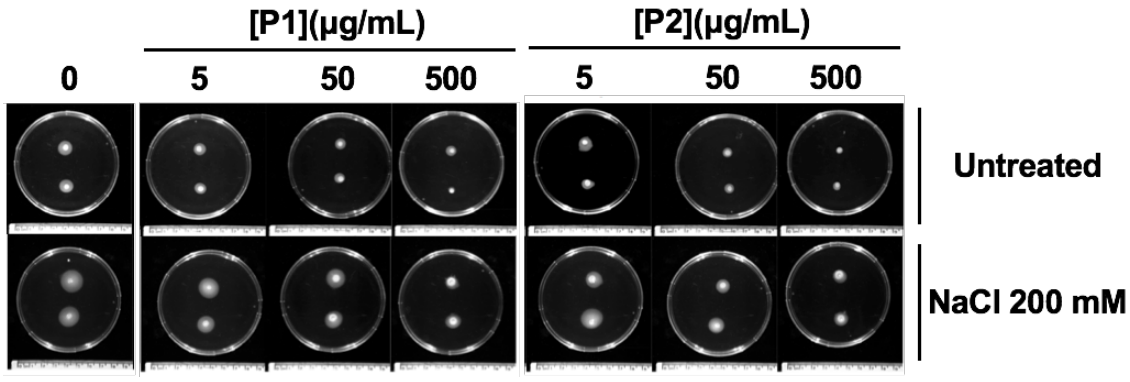

Fig. S4. Motility of *V. cholerae* N16961 in soft agar plates in the absence or presence of **P1**, **P2** and 200 mM NaCl.

9. Effect of polymers on microbial growth

9.1. GFP expression

Overnight cultures of GFP-expressing *V. cholerae* (as described in section 5) were diluted into DMEM without phenol red containing 50 µg/µL of spectinomycin to an OD_{600} of 0.02 as starting density. Polymers aliquots in DMEM were then added to give the desired final concentrations in 200 µL culture using a 96-well plate. The plate was covered with a BEM-1 breathe easy gas permeable membrane to avoid evaporation and incubated at 37 °C with constant shaking at 200 rpm. GFP fluorescence was recorded every 30 minutes over 24 hours using a FLUOstar Omega plate reader.

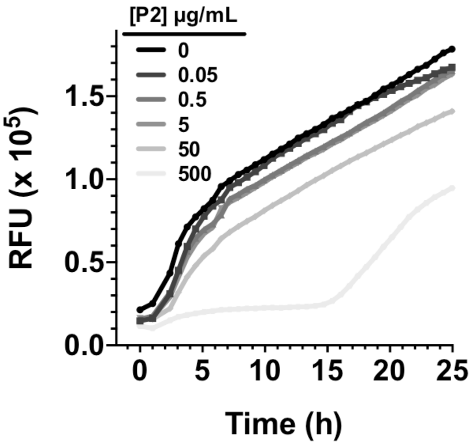

Fig. S5. Relative Fluorescent Units (RFU) for GFP expressing *V. cholerae* N16961 in the absence and presence of increasing amounts of **P2**. Initial OD_{600} = 0.02. Results are means ± s.e.m. of three independent experiments.

9.2. Microbial membrane integrity

Membrane integrity of *V. cholerae* cells was assessed by FACS using the LIVE/DEAD *Bac*Light™ kit (LifeTechnologies). Overnight cultures (as described in section 5) were diluted to an OD_{600} of 1.0 in 1 mL of DMEM without phenol red, and containing polymers at final concentrations ranging from 0 to 500 µg/mL and incubated for 20 hours. Following incubation, samples were stained according to the manufacturer's instructions. Readings were taken on an Attune Flow Cytometer, at a flow of 100 µL/min counting to 10,000 events. Samples containing no polymer were used as "non-permeable/viable" controls. In the case of "high permeability/dead" controls, DMEM was replaced by 70% 2-propanol and incubated for 1 h. Prior to staining, 2-propanol was removed and cells were washed once and resuspended in fresh DMEM to carry out the staining procedure.

10. Virulence

10.1. Quantification of biofilm formation

The amount of biofilm was determined by crystal violet staining, as previously described.[8] Briefly, an overnight culture of *V. cholerae* (as described in section 5) was diluted to an OD_{600} of 0.2 in fresh DMEM media and incubated in a 96-well plate overnight (i.e. 20 h) at 37°C with shaking at 200 r.p.m., in the presence and absence of polymers. Cultures were then removed and biofilm was rinsed with PBS and stained with 200 µL of 1% crystal violet solution in water. After 30 minutes, the crystal violet solution was removed and the well was rinsed again with PBS. 200 µL of 95% ethanol was then added to each well to detach the dye. The amount of biofilm was determined by measuring at 595 nm using a FLUOstar Omega plate reader.

10.2. Imaging of biofilm formation and quantification of extracellular DNA

Biofilms of GFP-*V. cholerae* were grown, as described above, for 20 hours at 37°C in 96-well glass-bottom plates containing bacteria at an initial OD_{600} of 0.2 in DMEM only, or DMEM containing **P1** or **P2** at concentrations ranging from 5 to 500 µg/mL, in a total volume of 200 µL. In the case of DNAase I treated samples, 2 units of DNAseI (NEB) were added and cultures incubated for another 2 hours at 37 °C. Following incubation, plates were rinsed with PBS, samples fixed with 4% formaldehyde in PBS for 15 minutes and then washed with PBS. DNA was stained with 10 µg/mL Hoechst in PBS for 10 minutes. Samples were imaged using a Zeiss Axio Observer.Z1 microscope, Zeiss 40x/1.4 Plan Apochromat,objective, ORCA-Flash4.0 camera (Hamamatsu) and ZEN 2.0.0.10 software. Images were processed using Image J and Corel Draw X5. Extracellular DNA was determined from the relative pixel intensity of blue channel from pictures taken with a Nikon TE2000-U microscope using a 100x Plan Apo objective (Nikon) and captured with Digital Sight DS-Qi1MC camera (Nikon). From each picture, the intensity of pixels was measured from the blue channel using ImageJ.

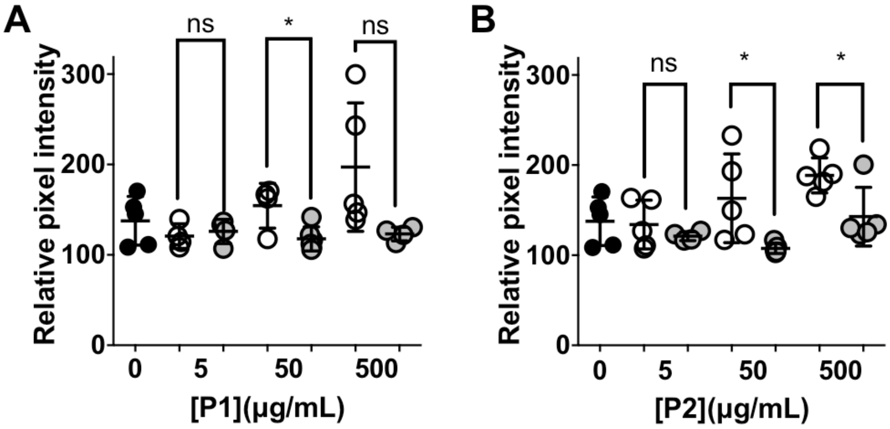

Fig. S6. Intensity of pixels from the blue channel from confocal micrographs of GFP expressing *V. cholerae* N16961 cultures in the absence (●) and presence of **P1** (**A**) or **P2** (**B**) (○). Polymer-induced clusters were also treated with DNAse I (●). Data are individual measurements from at least four representative images, means and stdev. Analysis of variance (ANOVA), followed by Tukey's post hoc test, was used to test for significance. Statistical significance was defined as p<0.05 (*).

10.3. Transcriptional assays: β-galactosidase activity

Overnight cultures of *V. cholerae* reporter strains were grown in LB at 37 °C, as described in section 5. Prior to infection, cultures were adjusted to an MOI of 10 and incubated at 30 °C for 1 hour in 1 mL of DMEM without phenol red, in the absence and presence of polymers (500 µg/mL **P1** and 50 µg/mL **P2**). Caco-2 cells were washed with PBS to remove media containing antibiotics and infections were started by transferring the above cultures of bacteria into the wells. Plates were centrifuged at 1000x g for 5 min at 20 °C to synchronise the infections. After 7 hours of incubation at 37 °C under 5% CO_2, Caco-2 were washed with PBS and homogenized with 1 mL of 0.5% triton X100 in PBS for 5 minutes. β-galactosidase activity was assayed as previously described.[9] Briefly, ortho-nitrophenyl-β-galactoside (ONPG) was dissolved at a final concentration of 0.2 mM in buffer Z with 0.27% of 2-mercaptoethanol. Bacterial cells were disrupted adding a drop of toluene and a drop of 1% sodium deoxycholate letting the tube open after vortexing for about 30 minutes to let the toluene evaporates at room temperature. 100 µL of the lysis solution were added with 2.5 mL of ONPG solution and time was recorded. When the solution turns yellow, 1 mL of 1M Na_2CO_3 was added to stop the reaction. Absorbance at 420 nm was recorded using a Jenway 6305/UV Visible spectrophotometer. Transcriptional activity (in Miller Units) was calculated as previously described.[9] Percentage reduction was calculated as the percentage difference between miller units in the absence and presence of the polymer.

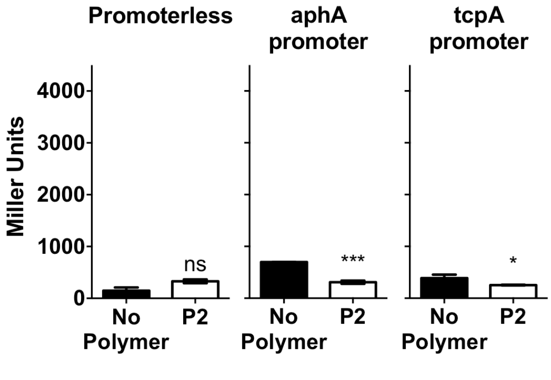

Fig. S7. Promoter activities of *aphA*- and *tcpA-lacZ* fusions following infection of Caco-2 cells for 7 hours in the absence (black) or presence of 50 µg/mL **P2** (hollow). Student's paired t-test was used to test for significance. Statistical significance was defined as p<0.05 (*) or p<0.005 (***). For consistency, the range of miller units is the same as the one used in the other transcriptional assays (**Fig. 4B** and **Fig. 5H)**.

11. Colonisation and toxicity of Caco-2 cells

11.1. Colony forming units

Overnight cultures of *V. cholerae* N16961 were grown in LB at 37 °C, as described in section 5. Prior to infection, cultures were adjusted to an MOI of 10 and incubated at 30 °C for 1 hour in 1 mL of DMEM without phenol red, in the absence and presence of polymers. Caco-2 cells were washed with PBS to remove media containing antibiotics and infections were started by transferring the above cultures of bacteria into the wells. Plates were centrifuged at 1000x g for 5 min at 20 °C to synchronise the infections. After 7 hours of incubation at 37 °C under 5% CO_2, Caco-2 were washed with PBS and homogenized with 1 mL of 0.5% triton X100 in PBS for 5 minutes. Once the debris was dislodged from the well, samples were serial diluted and plated out onto TCBS agar. Plates were incubated at 37 °C overnight for CFU counting.

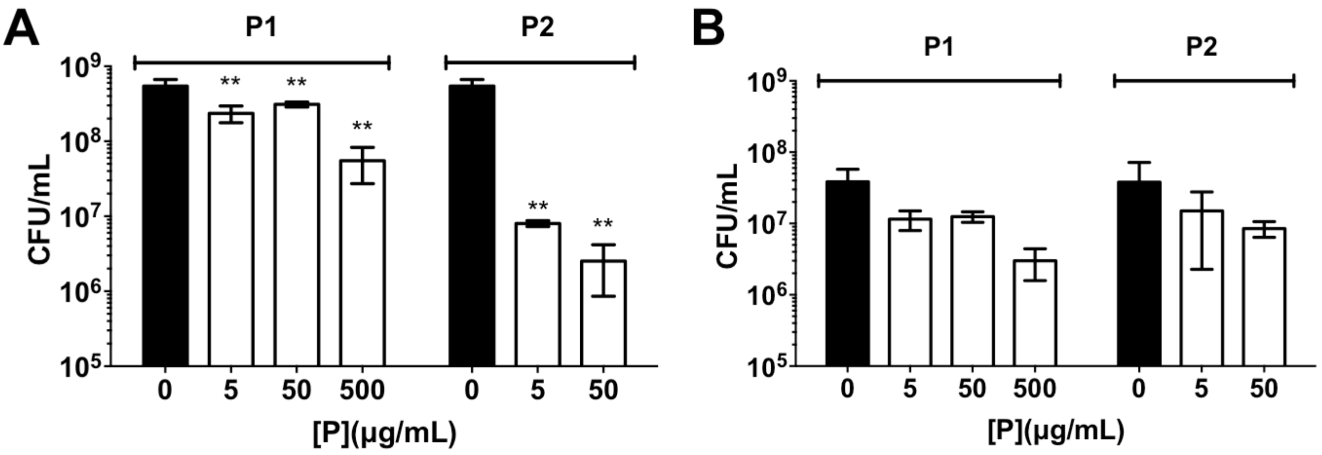

Fig. S8. Number of colony forming units per mL (CFU/mL) of GFP expressing *V. cholerae* N16961 (**A**) or BH1651 mutant (**B**) from Caco-2 cells incubated in the absence and presence of polymer treated *V. cholerae* cultures, following washing and lysis of Caco-2 cells. Results are means ± s.e.m. of three independent experiments. Analysis of variance (ANOVA), followed by Tukey's post h test, was used to test for significance. Statistical significance was defined as p<0.05 (*), p<0.01 (**) or p<0.0001 (****).

11.2. Cytotoxicity in Caco-2 cells: LDH assay

Overnight cultures of the GFP-expressing *V. cholerae* were grown in LB at 37 °C, as described in section 5. Prior to infection, cultures were adjusted to an MOI of 10 and incubated at 30 °C for 1 hour in 1 mL of DMEM without phenol red, in the absence and presence of polymers. Caco-2 cells were washed with PBS to remove media containing anti-biotics and infections were started by transferring the above cultures of bacteria into the wells. Plates were centri-fuged at 1000x g for 5 min at 20 °C to synchronise the infections. After 7 hours of incubation at 37 °C under 5% CO_2, supernatants were used to measure lactate dehydrogenase (LDH) activity using LDH Cytotoxicity Detection Kit (Takara Clonetech) according to the manufacturer's instructions. The data was expressed as percentage of Extracellular LDH, normalised to untreated cells (0%) and 0.1% Triton X-100 lysed cells (100% lysis) and was calculated according to **Equation 1**:

$$\textbf{Equation 1: } \%Extracellular\ LDH = \frac{OD_{490} - OD_{490}(Untreated)}{OD_{490}(Triton) - OD_{490}(Untreated)}$$

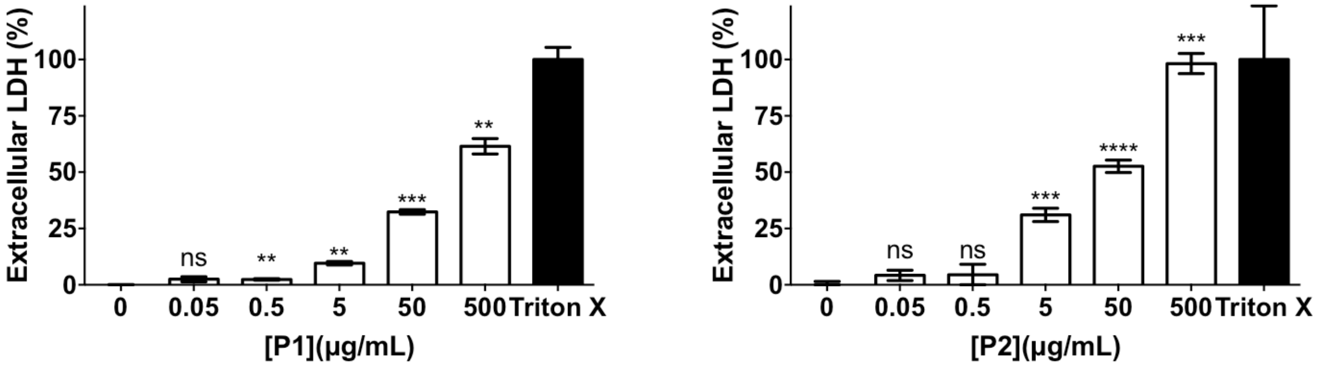

Fig. S9. Percentage of extracellular LDH in Caco-2 cultures incubated in the absence and presence of **P1** and **P2**. Results were normalised to untreated Caco-2 cells (0%) and cells lysed with Triton X-100 (100%). Results are means ± s.e.m. of three independent experiments. Analysis of variance (ANOVA), followed by Tukey's post hoc test, was used to test for significance. Statistical significance was defined as p<0.01 (**), p<0.001 (***) or p<0.0001 (****).

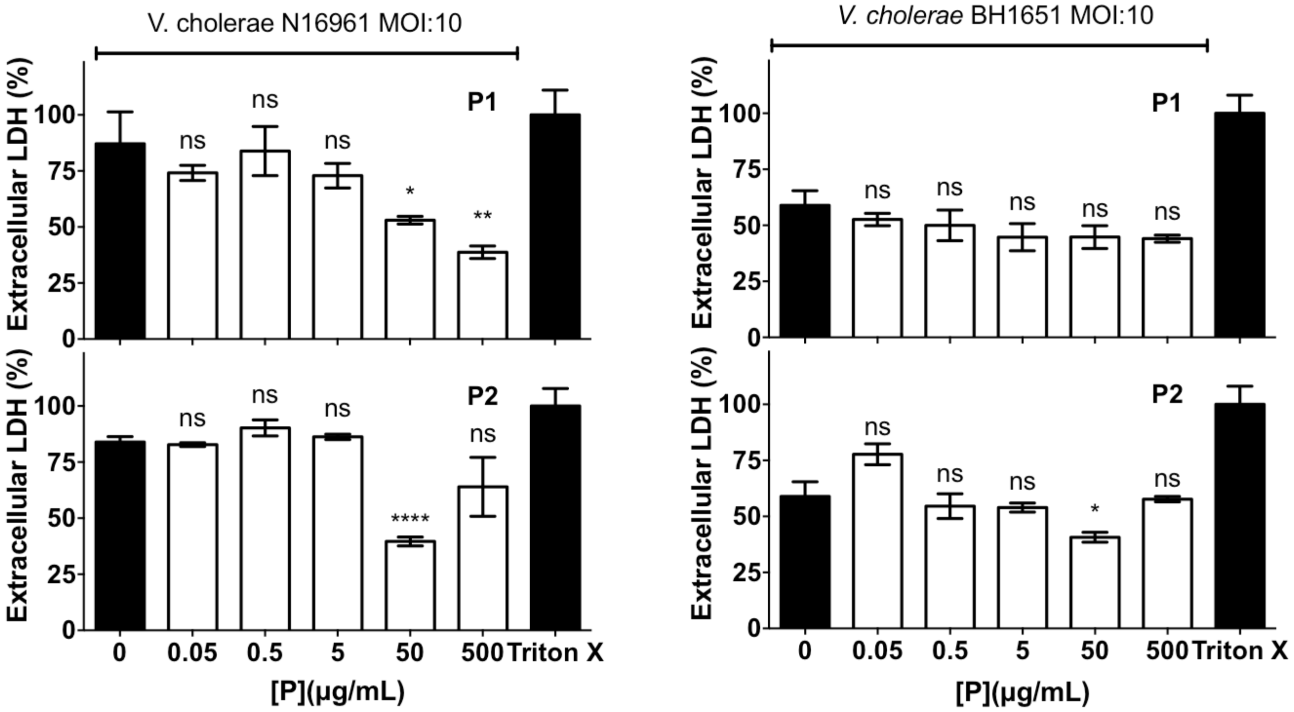

Fig. S10. Percentage of extracellular LDH in Caco-2 cultures incubated in the absence and presence of polymer treated *V. cholerae* N16961 cultures (**left**) or BH1651 mutant (**right**). Results were normalised to untreated Caco-2 cells (0%) and cells lysed with Triton X-100 (100%). Results are means ± s.e.m. of three independent experiments. Analysis of variance (ANOVA), followed by Tukey's post hoc test, was used to test for significance. Statistical significance was defined as p<0.05 (*), p<0.01 (**) or p<0.0001 (****).

11.3. Cytotoxicity in Caco-2 cells: cAMP assay

Overnight cultures of *V. cholerae* were grown in LB at 37 °C, as described in section 5. Prior to infection, cultures were adjusted to an MOI of 10 and incubated at 30 °C for 1 hour in 1 mL of DMEM without phenol red, in the absence and presence of polymers. Caco-2 cells were washed with PBS to remove media containing antibiotics and infections were started by transferring the above cultures of bacteria into the wells. Plates were centrifuged at 1000x g for 5 min at 20 °C to synchronise the infections. After 7 hours of incubation at 37 °C under 5% CO_2, supernatants were used to measure sample using Cyclic AMP Competitive ELISA kit (Invitrogen) following manufacturer´s instructions. Concentration of cAMP, expressed in pmol/mL, was estimated from a calibration curve made with pure cAMP.

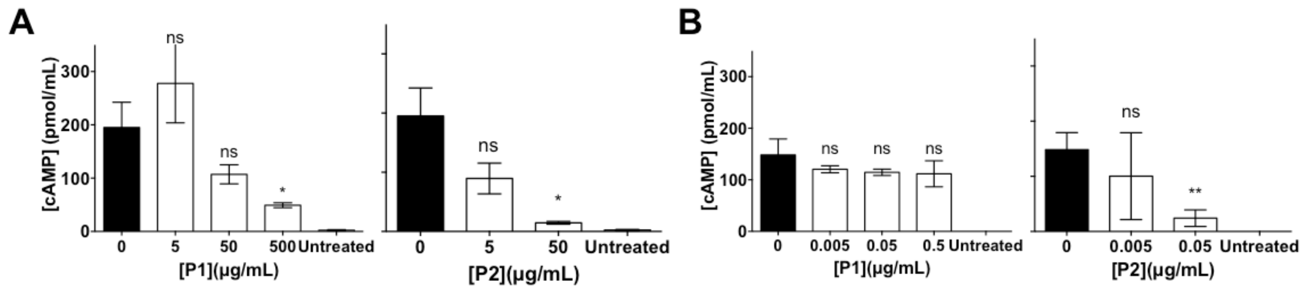

Fig. S11. Levels of cAMP production in Caco-2 cells in the absence and presence of polymer treated *V. cholerae* N16961 (**A**) or BH1651 mutant (**B**) cultures. *V. cholerae* cultures were adjusted to an MOI of 10, and incubated in the absence or presence of polymers for 1 h prior to infection of cultured Caco-2 intestinal epithelial cells for 7 hrs. Results are means ± s.e.m. of three independent experiments. Analysis of variance (ANOVA), followed by Tukey's post hoc test, was used to test for significance. Statistical significance was defined as $p<0.05$ (*), $p<0.01$ (**) or $p<0.0001$ (****).

11.4. Imaging of Caco-2 cells

Caco-2 cells were seeded onto sterilised glass cover slips inserted into the wells of the plate and incubated in the absence or presence of *V. cholerae* as described above. Imaging of infected Caco-2 cells was then done by fixing the samples with 4% formaldehyde in PBS for 15 minutes and then washing with PBS. Cells were permeabilised by adding 0.1% Triton X-100 in PBS and incubation at room temperature for 5 minutes, and washed three times with PBS. Samples were then stained with 10 µg/mL Hoechst and 66 ng/mL of rhodamine-phalloidin in the dark for 10 minutes to visualise DNA and F-actin, respectively. Staining was followed by three washing steps with PBS (5 minutes each). Samples were mounted using antifade gold mounting solution (Life-Technologies) and cured overnight at 22 ^{o}C prior to visualisation. Samples were viewed under a Zeiss Axio Observer.Z1 microscope with 63x/1.4 Plan Apochromat objective. Images were taken using Zen Pro software (Zeiss) and processed using ImageJ software.

12. Colonisation of Zebrafish

12.1. Zebrafish care and maintenance

Zebrafish (*Danio rerio* wild type strain AB) were kept in a recirculating tank system at the University of Birmingham Aquatic Facility. Zebrafish were kept under a 14 h-10 h light-dark cycle with water temperature maintained at 28 ^{o}C. Zebrafish care, breeding and experiments were performed in accordance with the Animal Scientific Procedures Act 1986, under Home Office Project License 40/3681. After collection of eggs, larvae were kept in a diurnal incubator under a 14 h-10 h light-dark cycle with the temperature maintained at 33 ^{o}C. Eggs were maintained at 40 eggs per 50 ml in E3 media plus 0.00003% methylene blue for 8 h and E3 media plus 26.6 µg/ml 1-phenyl-2-thiourea (PTU) to inhibit melanisation. The fish line used was wild-type AB zebrafish. All zebrafish care and husbandry procedures were performed as described previously.[10]

12.2. Colony forming units

Prior to infection, 10^6 and 10^7 CFU/mL of *V. cholerae* were incubated in 3 mL of E3 medium in the presence and absence of polymers. After 1 hour of incubation to ensure cluster formation, 2 mL of supernatant were removed from these cultures and replaced with 2 mL of E3 medium or 2 mL of E3 medium containing zebrafish larvae (5 days post fertilisation – d.p.f). Additionally, the 2 mL of supernatant were mixed with 3 mL of E3 medium or 3 mL of E3 medium containing zebrafish larvae (5 days post fertilisation – d.p.f), to evaluate the ability of unclustered bacteria to colonise the fish. *V. cholerae* cultures were then incubated with rotation at 25 ^{o}C for 6 hours. Larvae were then euthanised with an overdose of Tricaine-S (1600 µg/mL) and homogenised by washing the larvae with PBS and incubating them in 1% Triton X-100 for 30 minutes. Lysates were passed several times through a needle to homogenise and 100 µL of the resulting solution, as well as serial dilutions, were plated onto TCBS agar and colonies counted following overnight incubation at 37 ^{o}C.

12.3. Imaging of zebrafish larvae

Visualisation of zebrafish larvae was done by directly mounting the larvae in 0.4% low melting point agarose containing 160 µg/mL of Tricaine-S. Samples were viewed under a Zeiss Axio Observer.Z1 microscope with 63x/1.4 Plan Apochromat objective. Images were taken using Zen Pro software (Zeiss) and processed using ImageJ software.

13. References

1. J. B. Goldberg and D. E. Ohman, *J. Bacteriol.*, 1984, **158**, 1115-1121.
2. J. F. Heidelberg, J. A. Eisen, W. C. Nelson, R. A. Clayton, M. L. Gwinn, R. J. Dodson, D. H. Haft, E. K. Hickey, J. D. Peterson, L. Umayam, S. R. Gill, K. E. Nelson, T. D. Read, H. Tettelin, D. Richardson, M. D. Ermolaeva, J. Vamathevan, S. Bass, H. Qin, I. Dragoi, P. Sellers, L. McDonald, T. Utterback, R. D. Fleishmann, W. C. Nierman, O. White, S. L. Salzberg, H. O. Smith, R. R. Colwell, J. J. Mekalanos, J. C. Venter and C. M. Fraser, *Nature*, 2000, **406**, 477-483.
3. J. M. Ritchie, H. Rui, X. Zhou, T. Iida, T. Kodoma, S. Ito, B. M. Davis, R. T. Bronson and M. K. Waldor, *PLoS Pathogens*, 2012, **8**, e1002593.
4. W. L. Ng, L. Perez, J. P. Cong, M. F. Semmelhack and B. L. Bassler, *Plos Pathogens*, 2012, **8**.
5. J. Lodge, R. Williams, A. Bell, B. Chan and S. Busby, *FEMS Microbiol Letters*, 1990, **55**, 221-225.
6. D. H. Figurski, R. F. Pohlman, D. H. Bechhofer, A. S. Prince and C. A. Kelton, *Proceedings of the National Academy of Sciences of the United States of America*, 1982, **79**, 1935-1939.
7. M. Moisi, C. Jenul, S. M. Butler, A. New, S. Tutz, J. Reidl, K. E. Klose, A. Camilli and S. Schild, *J. Bacteriol.*, 2009, **191**, 7027-7038.
8. G. A. O'Toole, *Journal of Visualized Experiments*, 2011, DOI: 10.3791/2437.
9. A. I. Bell, K. L. Gaston, J. A. Cole and S. J. Busby, *Nucleic Acids Research*, 1989, **17**, 3865-3874.
10. M. Westerfield, *The Zebrafish Book*, University Press Oregon, Eugene, OR, 2000.
